# Variability of Sialic Acids in Beef Breeds and Nutritional Implications in Red Meat

**DOI:** 10.3390/molecules30030710

**Published:** 2025-02-05

**Authors:** Michela Contò, Maria Miarelli, Sabrina Di Giovanni, Sebastiana Failla

**Affiliations:** Consiglio per la Ricerca in Agricoltura e l’Analisi dell’Economia Agraria (CREA), Research Centre for Animal Production and Aquaculture, Via Salaria, 31, 00016 Monterotondo, Italy; michela.conto@crea.gov.it (M.C.); sabrina.digiovanni@crea.gov.it (S.D.G.)

**Keywords:** sialic acid, N-acetylneuraminic acid, beef breed, red meat, polar amino acids

## Abstract

This study examines the variability of sialic acids, specifically N-acetylneuraminic acid (Neu5Ac) and N-glycolylneuraminic acid (Neu5Gc), in beef from seven cattle breeds (Holstein Friesian, Red Pied, Maremmana, Chianina, Charolais, Limousin, and Piemontese). Neu5Gc, a non-human sialic acid linked to inflammation and disease risk, showed significant breed differences (*p* < 0.001), with the highest concentration in Holstein Friesian (61.02 µg/g) and the lowest in Piemontese (20.87 µg/g). Neu5Ac, known for its neuroprotective properties, was most abundant in Piemontese (112.99 µg/g, *p* = 0.032) and lowest in Limousin (81.25 µg/g). The Neu5Ac/Neu5Gc ratio, critical for dietary health, exceeded the threshold of 5:1 only in Piemontese (5.49), identifying it as a breed with a higher ratio. This study highlights the influence of breed, with limited effects of muscle type and aging, on sialic acid content. Significant correlations were observed between Neu5Gc and fatty acid classes (*p* < 0.05) and between Neu5Ac and polar amino acid groups (*p* < 0.01). The findings support selective breeding to optimize beef’s nutritional profile, enhancing its health benefits for consumers.

## 1. Introduction

Sialic acids, a heterogeneous family of nine-carbon sugars, are integral components of glycan structures on cell surfaces. As terminal residues on these glycan chains, they play a role in modulating protein stability, molecular recognition, and intercellular communication. The electronegative charge of sialic acids contributes to their functional specificity and interactions, playing crucial roles in various physiological processes [1,2,3,4]. A key aspect of sialic acid function is its interaction with immune receptors, particularly sialic acid-binding immunoglobulin-like lectins (Siglecs). These interactions mediate immune cell signaling, inflammatory responses, and pathogen recognition, leading to distinct biological effects [5].

Among the sialic acids, N-acetylneuraminic acid (Neu5Ac) and N-glycolylneuraminic acid (Neu5Gc) are the most prominent, exhibiting structural similarities and distinct biological roles. The structural difference between Neu5Ac and Neu5Gc arises from a single oxygen atom (Neu5Gc contains the glycolyl group -COCH_2_OH, compared to Neu5Ac, characterized by the acetyl group -COCH_3_), influencing their biochemical properties and biological roles. Neu5Ac is the predominant sialic acid in humans and vertebrates.

In humans, Neu5Ac plays a crucial role in immune modulation and protective physiological functions [3], and is essential for the nervous system by enhancing ganglioside functionality, promoting synaptic plasticity, neuroprotection, and signal transduction [6,7]. In contrast, Neu5Gc is common in most mammals but absent in humans. This absence is due to the evolutionary loss of the CMAH gene, which encodes CMP-N-acetylneuraminic acid hydroxylase, the enzyme responsible for converting Neu5Ac into Neu5Gc [2,8,9]. Humans, therefore, acquire Neu5Gc exclusively from dietary sources, primarily red meat. The absorption of sialic acids from animal products occurs through macropinocytosis from the intestine into lysosomes. This process predominantly involves conjugated sialic acids, while free sialic acids, which constitute only a minor fraction, are either directly utilized by the gut microbiome or excreted [2,10].

The incorporation of Neu5Gc from animal products into human tissues has been associated with potential health risks [11,12], particularly due to the production of xenoreactive anti-Neu5Gc antibodies [13,14]. This immune response can trigger xenosialitis, a chronic inflammatory process that may contribute to the progression of cardiovascular diseases, neurodegenerative disorders, and cancer.

However, despite some murine studies with human-like Neu5Gc-deficient *Cmah*-null mice and in vitro studies indicating that Neu5Gc accumulation correlates with a dose-dependent increase in carcinoma incidence [14,15,16], clinical evidence in humans remains limited. The large-scale NutriNet-Santé study [17] yielded controversial results, failing to establish a direct link between Neu5Gc intake and cancer risk.

The concentration of Neu5Gc in red meat varies significantly across studies [18], due to differences in experimental designs, methodologies, and analytical approaches. Several studies report data from single samples [19,20,21], limiting broader conclusions. In contrast, studies by Jahan et al. [22,23] analyzed multiple meat samples, reporting Neu5Ac levels ranging from 140 µg/mg protein in tenderloin to 392.29 µg/mg protein in neck muscles, while Neu5Gc concentrations varied from 30.97 µg/mg to 76.94 µg/mg protein across different cuts of lamb. These findings underscore the variability of sialic acid content between meat cuts and the critical need for standardized methodologies to enable accurate comparisons across studies and species. Furthermore, little is known about the variability in sialic acid content among different breeds within the same species [24].

Genetic polymorphisms may also influence sialic acid biosynthesis in cattle breeds, as suggested by Crisà et al. [25] for milk sialic composition. For instance, bovine breeds with reduced Neu5Gc levels in milk may produce meat with a lower Neu5Gc content.

Additionally, antioxidant-rich diets and low-stress farming conditions could improve sialic acid composition by modulating the animal’s immune system [26].

This variability could also be influenced by farming systems or processing methods, particularly when proteolytic activity during aging and conservation alters glycoproteins and glycolipids. Enzymatic degradation during aging increases the availability of free sialic acids, although the mechanisms remain unclear [24,26]. Quantifying the dietary intake of Neu5Gc and Neu5Ac through red meat consumption presents a challenge due to its significant variability, and because emerging research suggests that Neu5Ac may modulate Neu5Gc absorption.

Bardor et al. [27] demonstrated in cultured cells that Neu5Ac can metabolically compete with Neu5Gc for incorporation in human tissue. They concluded that a Neu5Ac concentration at least five times higher than Neu5Gc could significantly limit the intestinal absorption of Neu5Gc. In a subsequent study [28], the same authors investigated whether a similar competitive mechanism between these two sialic acids could act in a murine model of atherosclerosis, potentially reducing xenosialitis-driven inflammation and its contribution to atherogenic plaque development. To test this hypothesis, they provided human-like *Cmah*-null mice with six different diets over 12 weeks. Two diets were enriched exclusively with Neu5Gc derived from red meat or Neu5Ac derived from edible bird nests, rich exclusively in this sialic acid. Another diet maintained a Neu5Ac to Neu5Gc ratio of 5:1, administered continuously for 12 weeks. The remaining three diets alternated between Neu5Ac and Neu5Gc, splitting the 12-week period into two phases (4 weeks and 8 weeks) with varying compositions. Atherosclerotic plaques in the aortic sinus of mice were assessed using anti-CD68 immunostaining.

The results showed that mice on the Neu5Ac only diet or the 5:1 Neu5Ac to Neu5Gc diet exhibited no necrotic cores and eliminated atherosclerotic plaques. In contrast, necrotic plaques were prominent in animals fed exclusively with Neu5Gc. This is likely due to Neu5Ac and Neu5Gc, derived from the same meal, entering cells simultaneously and competing for the same transporter and lysosomal sialidase.

If validated in humans, this competitive mechanism could limit the pro-inflammatory effects of Neu5Gc, which appear to be dose-dependent [29]. These findings emphasize the importance of evaluating not only the absolute levels of Neu5Gc in red meat but also the Neu5Ac to Neu5Gc ratio. This approach may offer a more comprehensive understanding of the potential health impacts of meat consumption [28].

Beef from breeds with lower Neu5Gc levels or higher Neu5Ac to Neu5Gc ratios could provide a healthier alternative for consumers concerned about chronic inflammation and the associated health risks [27,28].

Some authors, as reported in Liang et al. [26], have explored biochemical methods to inhibit Neu5Gc synthesis. Specifically, researchers have focused on blocking the activity of sialyltransferase, the enzyme responsible for converting free Neu5Gc into bound Neu5Gc. Li et al. [30] studied the effect of dietary supplementation with 5′-cytidine monophosphate (5′-CMP) on Neu5Gc content in Xiang pigs. Over 30 days, pigs supplemented with 5′-CMP showed a 58.18% reduction in Neu5Gc in muscle tissue. The study also reported significant changes in the free amino acid profile, including an increase in histidine content. The inhibition of sialyltransferase activity suggests a novel strategy to reduce Neu5Gc levels in red meat prior to slaughter.

Additionally, though numerous studies suggest a link between Neu5Gc consumption and an increased risk of cancer, metabolic diseases, and degenerative conditions [10], this hypothesis is beginning to be partially contradicted by recent research. Although Neu5Gc is considered an exogenous sialic acid for humans, there are hypotheses that humans may be able to produce Neu5Gc endogenously under certain conditions [31].

Specifically, in hypoxic cellular environments, a metabolic pathway involving high levels of reactive oxygen species (ROS) could convert Neu5Ac into Neu5Gc. This process is particularly relevant in inflammatory and cancerous tissues, where ROS levels are elevated. Furthermore, as reported by Bai et al. [31], in vitro studies using A549 cell cultures demonstrate that, in the presence of Neu5Ac, H_2_O_2_, and FeCl_2_, Neu5Gc can be generated from Neu5Ac without relying on the enzymatic pathway, dependent on the concentrations of iron and H_2_O_2_. To provide further evidence that Neu5Gc can be converted from Neu5Ac in the presence of oxidative factors, isotope-labelled Neu5Ac was applied in the buffer reaction, confirming the nonenzymatic conversion to obtain the glycolylneuraminic form of sialic acid. These findings open new avenues for research on the potential role of red meat in promoting inflammation and strategies to address the associated health concerns.

Addressing these issues could form the basis for dietary recommendations and farming practices that support healthier meat consumption. Such strategies should balance the essential nutrients found in animal-derived products (e.g., essential amino acids, fatty acids, B vitamins, particularly B12, and bioavailable minerals such as zinc and iron) [13,32] with the need to minimize the health risks associated with Neu5Gc.

The aim of this study is to evaluate the variability of Neu5Gc and Neu5Ac concentrations and their ratios among different cattle breeds, muscle types, and aging periods. This research is unique in highlighting breed-specific variability within a single experiment. Furthermore, the dietary role of Neu5Ac in modulating Neu5Gc absorption and incorporation into human tissues warrants detailed investigation, given the significant role of meat in the human diet and its high nutritional value. Finally, the sialic acid composition of beef should be systematically evaluated to optimize both its nutritional quality and the health outcomes for consumers.

## 2. Results and Discussion

The cattle breeds studied in this experiment can be classified into four main categories based on production purpose: dairy, dual purpose, rustic, and high muscularity breed meat [33]. The breeds used are summarized below. The Italian Holstein Friesian (HF) is one of the most important dairy breeds, and the meat of the young bull is widely commercialized despite lacking morphological traits [34]. The Italian Red Pied (RP) is considered a dual-purpose breed, producing good quantities of milk but also meat [35]. Rustic breeds such as the Maremmana (MM) are adapted to extensive grazing systems and are key players in sustainable meat production. This breed is renowned for its ability to thrive in harsh environments [36]. The Chianina (CN) is one of the oldest cattle breeds, and was historically used as a draught animal but is now primarily reared for beef production. This breed is protected under the Protected Geographical Indication (PGI) “Vitellone Bianco dell’Appennino Centrale” [36,37]. Among cosmopolitan beef breeds, the Charolais (CH) and the Limousin (LM) originating from France were used. These breeds are valued for their large size, rapid growth, and ability to produce lean beef [38]. The double-muscled breed studied in this work was the Piedmontese (PD), characterized by high muscularity, a result from a mutation in the myostatin gene that causes muscle hypertrophy [34,38].

Each of these breeds plays a distinct role in the beef industries, contributing to both local food traditions and the international market.

### 2.1. Chemical Composition of Meat

#### 2.1.1. Fatty Acid Profile

The composition of fat and fatty acids (FAs) in meat is strongly influenced by diet but also by genetic factors. Typically, beef is characterized by a lower proportion of polyunsaturated fatty acids (PUFAs) due to the action of the rumen. Rumen bacteria facilitate the process of biohydrogenation, converting fatty acids such as linolenic acid into saturated fatty acids (SFAs) like stearic acid [39], resulting in higher SFA accumulation. This trend can be mitigated by implementing diets rich in PUFAs or using pasture-based feeding systems. In this study, the fat percentage (Table 1) of the *Longissimus thoracis* (LTm) muscle was generally low, particularly in the PD breed, which exhibited extremely lean meat with a fat content of 1.43%. Other breeds, such as the RP, LM, and MM, produced lean LTm, while the last three breeds displayed moderately higher fat percentages, averaging 2.73%. The increase in intramuscular fat content was also associated with a greater accumulation of SFAs, particularly in the HF breed (46.51%). In contrast, the LM and PD breeds exhibited a lower percentage of SFAs, with an average of 41.06% for the two breeds. The remaining breeds showed intermediate values, ranging from 45.72% to 43.58%. No significant differences were observed in the monounsaturated fatty acids (MUFAs). However, the PUFA content was notably higher in the PD breed, where PUFAs constituted 21.17% of the total fatty acids, a result consistent with the findings of Brugiapaglia et al. [34]. The PUFA percentages for the LM and CN breeds were not significantly different from those of the PD breed. In contrast, beef breeds such as the CH and dairy breeds like the HF and RP, which are typically fed diets rich in maize silage, exhibited the lowest PUFA content, averaging 13.46% for the three breeds. The MM breed displayed intermediate PUFA levels compared to other breeds.

A study conducted by Savane et al. [40], comparing 15 different breeds fed with an identical diet with a low fiber ratio, reported results consistent with those observed in this study. The Holstein breed had the highest SFA content, while the PD and LM breeds displayed the lowest SFA content and the highest PUFA percentage. These results support the hypothesis that genetic factors contribute to differences in fatty acid profiles, even when animals are raised under distinct conditions specific to their breed and geographic location.

#### 2.1.2. Amino Acid Composition

Breeds with higher fat content tended to exhibit a lower protein percentage (Table 1), as observed in the CN and HF breeds, which had the lowest average protein content (20.98% for both breeds). Conversely, breeds such as the PD and RP displayed higher protein percentages (22.33% on average). The remaining breeds fell within an intermediate range for protein content.

Amino acids, the fundamental building blocks of proteins, play a critical role in the nutritional and functional properties of beef. Beef is considered a high-quality protein source due to its balanced amino acid profile, which includes essential amino acids (EAAs) that cannot be synthesized by the human body. These amino acids contribute not only to the nutritional value of beef but also to its flavor, texture, and overall sensory characteristics [32]. Beef contains all the EAAs required for human growth, repair, and overall health, each with distinct biochemical properties and functions [41]. The nine EAAs are histidine (His), isoleucine (Ile), leucine (Leu), lysine (Lys), methionine (Met), phenylalanine (Phe), threonine (Thr), tryptophan (Trp), and valine (Val). The highest percentages of EAAs were recorded for the HF and CN breeds, but the differences were not significant due to great variability of the single amino acids, as displayed in Supplementary Table S1. Comparative studies on different cattle breeds, such as that by Vopálenský et al. [42], revealed few significant differences in EAA content, with noteworthy differences observed only in the levels of Val and Met. However, studies comparing cattle breeds in terms of amino acid content remain limited.

Among the EAAs, a specific subgroup, the branched-chain amino acids (BCAAs), including leucine (Leu), isoleucine (Ile), and valine (Val), was reported. In this study, beef was generally characterized by a high proportion of BCAAs. In particular, rustic Italian breeds, which were historically used as working animals, tended to accumulate higher levels of BCAAs (18.13% vs. 15.91%, respectively, for MM and CN compared to the others).

These amino acids are essential for energy metabolism, particularly during prolonged physical activity. They also support immune function by promoting immune cell production, which is particularly important during periods of stress or illness, as well as the synthesis of intestinal mucosal proteins [43]. BCAAs also influence glucose metabolism through their role in energy production and the regulation of insulin signaling [44]. Although BCAAs do not directly interact with sialic acids, they may indirectly affect the availability of essential precursors required for sialic acid synthesis.

Another important group of amino acids in beef are polar amino acids (PAAs), which play active roles in various metabolic processes. PAAs are typically classified into distinct subgroups. One contains hydroxyl groups, including serine (Ser), threonine (Thr), and tyrosine (Tyr). Another subgroup includes amino acids with carboxyl groups, such as aspartic acid (Asp) and glutamic acid (Glu). Additionally, cysteine (Cys), a polar amino acid containing a thiol group, plays a vital role in oxidative processes [45].

In the LTm, higher quantities of PAAs were observed in the HF, CN, and PD breeds, with an average of 40.33%. In contrast, the lowest PAA values were recorded in the MM (36.19 %) and LM breeds (35.69%). The remaining breeds displayed intermediate PAA percentages. Vopálenský et al. [42] also reported significantly lower levels of Thr and Ser in the LM breed. Dairy breeds typically have a higher proportion of polar amino acids due to the greater oxidative metabolism of their muscles.

Some of these amino acids are involved in the synthesis of mucin proteins, which are essential for maintaining intestinal integrity and function. They also play a critical role in physiological processes, such as protein phosphorylation and O-linked glycosylation, thereby actively modulating numerous biological functions. During glycosylation, sialic acids are often linked to specific glycans. In N-linked glycosylation, the glycan is attached to the nitrogen atom in the side chain of asparagine residues, while in O-linked glycosylation, glycans are attached to the oxygen atom in the side chains of serine or threonine residues [1]. These polar amino acids play a crucial role in protein glycosylation, where carbohydrates are attached to the hydroxyl (-OH) groups of these amino acids. This modification occurs primarily in the Golgi apparatus, affecting the structural and functional properties of glycoproteins and glycolipids. Among the most significant terminal modifications in glycosylation is sialylation, where sialic acids, both Neu5Ac and Neu5Gc, are added to the glycan chains [3]. Although the percentage of this amino acid and the sialic acid content is not directly correlated, it is interesting to note in the supplemental data that Ser and Thr are abundant in the HF breed. The relationship between Ser/Thr availability in tissue and Neu5Gc content is not yet fully understood.

### 2.2. Sialic Acid Content

#### 2.2.1. Sialic Acid Content in Seven Breeds

The sialic acid content observed in beef breeds is presented in Table 2. The data revealed significant differences across breeds, particularly for Neu5Gc.

The HF breed exhibited the highest Neu5Gc levels (61.02 µg/g), while the PD breed showed the lowest (20.87 µg/g), with a difference of approximately 40 µg/g. The LM breed also demonstrated low Neu5Gc levels, which were not significantly different from those of the PD breed. Intermediate values were observed in other breeds, with an average of 48.14 µg/g.

Neu5Ac levels were highest in the PD breed (112.99 µg/g) and lowest in the LM breed (81.25 µg/g). The HF breed displayed high Neu5Ac levels, comparable to those of the PD breed. The MM breed also presented notable Neu5Ac levels. Other breeds exhibited significantly lower Neu5Ac levels, ranging from 81.25 µg/g to 93.50 µg/g.

The Neu5Ac/Neu5Gc ratio varied significantly among the studied breeds. The PD breed had the highest ratio (5.49), surpassing the recommended dietary threshold of 5:1 proposed by Kawanishi et al. [16] to limit the absorption of Neu5Gc. The MM and LM breeds also exhibited relatively high ratios (3.48 and 3.15, respectively). The CN breed showed the greatest variability in the Neu5Ac/Neu5Gc ratio, with values ranging from 1.59 to 3.18. On average, most of the literature report Neu5Ac/Neu5Gc ratios of about 2.

The Neu5Ac/Neu5Gc ratios approached the ratio considered optimal, exceeding the value of 4 in only three samples from the MM breed, one sample from the LM breed, and five samples from the PD breed.

The mean values reported for each breed exhibited substantial variability within the breeds themselves, as illustrated in Supplementary Figure S1a,b.

Neu5Gc showed high variability between breeds (coefficient of variation, CV = 41.66%), while Neu5Ac exhibited lower variability (CV = 19.45%). Among breeds, the LM displayed the highest intraclass variability for both Neu5Gc and Neu5Ac.

Considering the data, only two meat samples from the MM breed and five samples from both the LM and PD breeds had Neu5Gc concentrations below 25 µg/g.

Different authors have analyzed sialic acids in beef, but most studies are limited by a small sample size [20,21,29,46], making it difficult to accurately assess data variability. Reported values for Neu5Gc in the literature range from 25 µg/g to 36 µg/g, while Neu5Ac values range from 46 µg/g, as reported by Chen et al. [20], to 78 µg/g, as reported by Samraj et al. [29]. In this study, the highest observed value for Neu5Ac was 153 µg/g in an HF breed animal, while the lowest was 65.2 µg/g in an LM breed animal.

Samraj et al. [29] incorrectly reported variability for Neu5Gc as ranging from 25 µg/g to 213 µg/g; however, the 213 µg/g value corresponds to cooked and subsequently freeze-dried meat, making it incomparable to the 25 µg/g found in raw meat.

Other authors [21,29] have highlighted the effects of cooking, noting an increase of approximately 40–50% for both Neu5Gc and Neu5Ac from raw to cooked meat. Liang et al. [26] hypothesized that frying meat at high temperatures (above 150 °C) could release free Neu5Gc, making it non-absorbable by humans.

Only a few studies have examined in beef the differences between conjugated and free sialic acids [20,22,23,29], and those studies have reported low levels of free sialic acids in meat. Most of the sialic acids in beef are in a conjugated form, which influences their absorption and bioavailability.

#### 2.2.2. Sialic Acid Content in Different Muscles and Ageing Times

**Figure 1:**
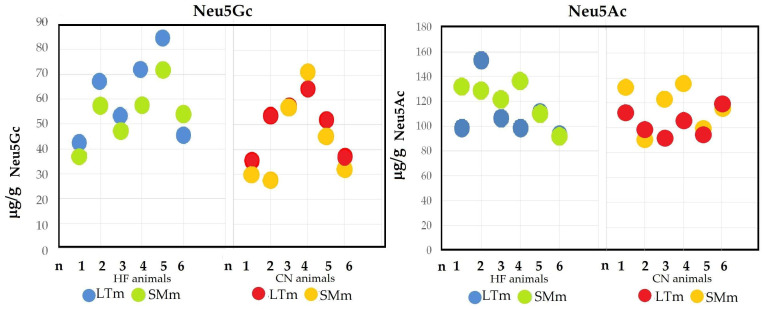
Content of N-acetylneuraminic acid (Neu5Ac) and N-glycolylneuraminic acid (Neu5Gc) in the *Longissimus thoracis* muscle (LTm) and *Semimembranosus* muscle (SMm) in six animals of the Italian Holstein Friesian (HF) breed and six animals of the Chianina (CN) breed.

No significant differences were observed for Neu5Gc (*p* = 0.353) or Neu5Ac (*p* = 0.124) between the two different muscles *Longissimus thoracis* (LTm) and *Semimembranosus* (SMm), as shown in Figure 1. The comparison was limited to the HF and CN breeds that exhibited the highest Neu5Gc levels and the lowest Neu5Ac/Neu5Gc ratios compared to the other breeds.

Among the six HF animals, five exhibited lower Neu5Gc levels in the SMm compared to the LTm, while one animal displayed lower Neu5Gc levels in the LTm. In the CN breed, Neu5Gc levels in the SMm were lower than those in the LTm for four animals, particularly for animal 2, while one animal exhibited higher Neu5Gc levels in the SMm, and another showed comparable levels in both muscles. The averages of the six animals are reported in Table S2.

For Neu5Ac, the differences were less pronounced. In HF animals, three displayed higher Neu5Ac levels in the SMm, two showed similar levels between the two muscles, and one exhibited lower Neu5Ac levels in the SMm. In CN animals, three displayed comparable Neu5Ac levels between the two muscles, while the remaining three had higher Neu5Ac levels in the SMm.

The ratio between the two sialic acids showed a different trend (*p* = 0.064) between the two muscles, particularly in the CN breed (2.07 vs. 2.65 for the LTm and SMm, respectively). Generally, the ratio increased when moving from the LTm muscle to the SMm muscle, as observed in the HF breed as well (1.81 vs. 2.21 for the two muscles, respectively).

There is limited literature concerning the differences in sialic acid content between lean muscles in beef. A study on different meat cuts focused on 10 commercial cuts of ovine carcasses [22,23]. Similar to our findings, no significant differences were found in the sialic acid content of meat from different regions of lamb carcasses with similar fat content.

**Figure 2:**
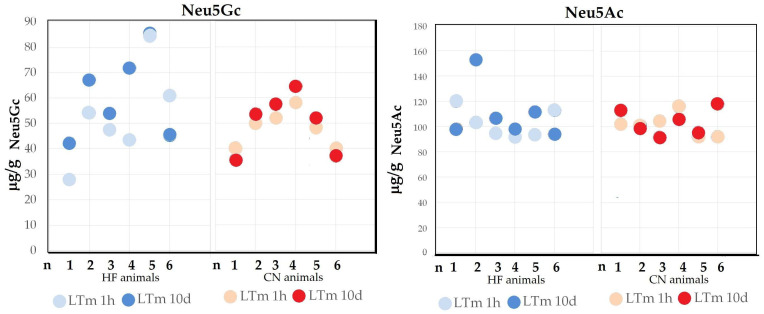
Content of N-acetylneuraminic acid (Neu5Ac) and N-glycolylneuraminic acid (Neu5Gc) in the *Longissimus thoracis* muscle (LTm) at 1 h and 10 days of aging in six animals of the Italian Holstein Friesian breed (HF) and six animals of the Chianina breed (CN).

Aging during meat processing did not show consistent effects on Neu5Gc or Neu5Ac levels (Figure 2).

Muscle sampling at different time points did not provide conclusive evidence of significant changes (Figure 2). Using the LTm muscle from the HF and CN breeds, samples were collected 1 h post-slaughter during the pre-rigor phase, and another set of samples was collected after 10 days of aging. The aging period did not reveal significant differences in sialic acid content for either breed (*p* = 0.408 for Neu5Gc and *p* = 0.485 for Neu5Ac).

For Neu5Gc, an increase was observed in four out of the six HF animals, with levels rising between 1 h post-slaughter and 10 days of aging. One HF animal exhibited lower Neu5Gc levels after aging, while another showed no substantial change. Similarly, in the CN breed, Neu5Gc levels increased in four animals after 10 days of aging compared to 1 h post-slaughter, while the remaining two animals displayed the opposite trend, with lower levels after aging.

Regarding Neu5Ac, its levels decreased in two HF and two CN animals after 10 days of aging. However, four HF animals exhibited an increase in Neu5Ac between 1 h post-slaughter and 10 days of aging. In the CN animals, two of the remaining four animals showed no significant differences, while the other two exhibited lower Neu5Ac levels at 10 days compared to the initial 1-h post-slaughter sampling.

The ratio between the two sialic acids did not show significant differences due to sampling time, with an average value of 1.98.

Our data did not reveal any significant reduction in Neu5Gc levels during the aging period of meat. This finding contrasts with other tenderization techniques, such as electrical stimulation, which have demonstrated positive effects on Neu5Gc reduction. Xu et al. [47] investigated the impact of electrical stimulation on Neu5Gc content in red meat. Their results showed that electrical stimulation at 120 V for 50 s reduced Neu5Gc content by 74.24%, while maintaining the same texture and color of the meat. Theoretical calculations suggested that Neu5Gc becomes highly unstable under electrical stimulation, leading to the dissociation of molecular bonds. This discovery highlights a promising method for reducing dietary exposure to Neu5Gc in humans.

The variability reported in this experiment is of critical importance as it offers an opportunity to implement selection strategies aimed at reducing the presence of Neu5Gc, a molecule widely associated with inflammation, colon cancer, and metabolic diseases. Additionally, it provides the potential to increase the availability of Neu5Ac, a sialic acid with notable nutraceutical properties, as reported by the EFSA [48].

The large variability reported suggests that genetic selection could be used to improve this trait, increasing Neu5Ac levels while reducing Neu5Gc. Another possible strategy involves silencing the CMAH gene, as has been done in pigs to produce organs suitable for transplantation [49].

For a balanced diet, it is recommended that the Neu5Ac/Neu5Gc ratio exceed 5:1, as Neu5Ac is associated with health benefits, while Neu5Gc is considered potentially harmful. Among the analyzed breeds, only the PD breed consistently meets this threshold, making it a promising option for healthier meat consumption.

Overall, beef still falls short of optimal sialic acid values compared to other meats such as poultry or rabbit, which lack Neu5Gc entirely [18].

#### 2.2.3. Relationship Between Sialic Acid and Chemical Characteristics

The correlation coefficients among sialic acids and chemical compositions in beef are reported in Table 3. Leaner breeds, such as the PD and LM, exhibit lower Neu5Gc levels, while HF, which has a higher fat percentage, shows a greater quantity of Neu5Gc. As a result, the breed effect and the fat effect are confounded, making it difficult to accurately assess the relative contribution of each factor. Additionally, animals with higher fat content are more likely to have higher levels of SFAs and lower levels of PUFAs. Consequently, the correlation with Neu5Gc is likely influenced by breed effects, as described previously.

Other studies have highlighted differences in sialic acid content based on the leanness or fatness of the meat. For instance, Karunanithi et al. [50] reported that lean meat contained 22 µg/g and 39.7 µg/g of Neu5Gc and Neu5Ac, respectively, while fatty meat had higher values of 31.2 µg/g and 50.8 µg/g for Neu5Gc and Neu5Ac, respectively. These values, although relatively low and moderately variable, align with the trends observed in this study, where breeds with higher fat content also displayed higher concentrations of both sialic acids.

Regarding proteins, the breed effect is similarly confounded. Animals with lower fat content typically exhibit higher protein percentages. For amino acid groups, there is a significant correlation between Neu5Ac and EAAs. Even more notable is the significant positive correlation (*p* < 0.01) with PAAs, where some amino acids play a key role in O- linked bonds [1,2,3]. Currently, there is no direct evidence to suggest a specific interaction between branched-chain amino acids (BCAAs) and sialic acids.

The relationship between the two sialic acids and the main chemical characteristics of muscles may depend on various factors related to the animals, the cellular environment, and other conditions. It may also influence the ability of sialic acids to form stable bonds with glycoproteins and glycolipids; however, much remains to be clarified in this process. Regarding the correlation between sialic acids and fat, positive correlations have been observed with both Neu5Gc and the Neu5Ac/Neu5Gc ratio. This relationship is supported by the findings reported in Samraj et al. [29], which indicate that bovine fat contains a higher concentration of sialic acids compared to lean meat.

## 3. Materials and Methods

The study was conducted on six *Longissimus thoracis* muscle samples (LTm), provided by farms associated with purebred livestock committees. The meat came from two dairy breeds, the Italian Holstein Friesian (HF) and the Italian Red Pied (RP), a rustic breed, the Maremmana (MM), and Chianina (CN) Italian traditional beef. Additionally included were two more French breeds, the Limousin (LM) and Charolais (CH), reared in Italy for beef production, and the Piemontese breed (PD), specialized meat breeds with hypertrophic muscle development.

For the HF and CN breeds, additional samples from the same animals were collected from a second muscle, the *Semimembranosus* (SMm). From these same two breeds, meat samples were also collected at two different post-mortem time points. One steak was taken from the 13th rib one hour post-slaughter (*pre-rigor mortis*), while a second steak was obtained from the 12th rib of the lumbar region after 10 days of aging.

From all animals, the LTm samples were collected from the 12th rib of the lumbar region after 10 days of aging. These samples were dissected and the core of each LTm (about 100 g) was cleaned and frozen immediately after collection, transported in liquid nitrogen, and stored at −80 °C at the CREA laboratory. Before analysis, the samples were thawed at room temperature for 8 h, subdivided into three parts, and homogenized in an ice bath using an Ultra-Turrax T25 (IKA-Werke GmbH & Co., KG, Staufen, Germany). The pH of the thawed samples was measured to confirm that the aging process had progressed correctly.

This comprehensive sampling strategy allowed for the evaluation of variability in meat quality across species, breeds, muscle types, and aging times analyses.

### 3.1. Proximate Composition

The proximate analyses of the LTm muscle were conducted following the procedures outlined by the AOAC [51]. The analysis included the determination of dry matter (DM) using method 934.01, crude fat (EE) using method 920.39, ash content using method 942.05, and crude protein (CP) using method 984.13.

### 3.2. Fatty Acids Profile 

Fat extraction was performed on approximately 10 g of a minced LTm sample using a chloroform–methanol mixture in a 2:1 (*v*/*v*) ratio. Fat was methylated using a 2M methanolic KOH solution to produce fatty acid methyl esters (FAMEs) in hexane. The quantification of FAMEs was carried out using gas chromatography (GC) on an Agilent 6890N system (Agilent, Inc., Santa Clara, CA, USA) equipped with a flame ionization detector (FID) and a CP-Sil88 fused silica capillary column (100 m × 0.25 mm internal diameter, 0.2-μm film thickness; Agilent Technologies). Details regarding the procedures for fatty acid extraction, methylation, and GC conditions are described in Cifini et al. [52]. Prior to fat extraction, an internal standard (C19:0) was added to each sample to facilitate quantification.

The identification of FAMEs was achieved by comparing the retention times of sample peaks with those of reference peaks from a Supelco 37-component FAME mix (Sigma-Aldrich Merck, Darmstadt, Germany). The fatty acids were classified into saturated fatty acids (SFAs) as a sum of 12:0, 14:0, 15:0, 16:0, 17:0, 18:0, 20:0, and 22:0; monounsaturated fatty acids (MUFAs) as a sum of 16:1, 18:1 cis 9, 18:1 cis 11, and trans isomers of 18:1, 20:1, and 22:1, and polyunsaturated fatty acids (PUFAs) as a sum of 18:2 n-6, 18:3 n-6, 18:3n-3, 20:2n-6, 20:3 n-3, 20:4 n-6, 20:5 n-3, 22:5 n-3, 22:6 n-3, and CLA. Each single fatty acid was expressed as a percentage of the total FAMEs.

### 3.3. Amino Acid Determination 

The total amino acid content was determined using the AccQ•Tag Fluor Reagent Kit (Waters, Milford, MA, USA), as outlined in the Waters manual. The analysis was conducted on an HPLC system (Waters Alliance 2695, Waters Corporation, Framingham, MA, USA) equipped with an AccQ•Tag column (3.9 × 150 mm, Waters Corporation, Framingham, MA, USA). Approximately 1 g of the sample was hydrolyzed using 6N HCl at 110 °C for 14 h. After hydrolysis, the samples were appropriately diluted and derivatized with the AccQ•Tag reagent. Detection was carried out using fluorescence with excitation at λEX = 205 nm and emission at λEM = 395 nm. Identification of amino acid peaks was achieved by comparing the sample peaks to those of reference standards from the Waters AccQ•Tag system. Single amino acids were expressed as a percentage of the total amino acids. The amino acids were classified as essential amino acids (EAAs) including histidine (His), isoleucine (Ile), leucine (Leu), lysine (Lys), methionine (Met), phenylalanine (Phe), threonine (Thr), and valine (Val); branch amino acids (BAAs) including Val, Ile, and Leu; and polar amino acids (PAAs) including serine (Ser), threonine (Thr), tyrosine (Tyr), aspartic acid (Asp), glutamic acid (Glu), and cysteine (Cys).

### 3.4. Sialic Analysis

The quantification of sialic acids (Neu5Ac and Neu5Gc) in meat samples was performed using a modified method based on the protocols of Hara et al. [53], as reported in Failla et al. [19]. A total of 1 g of homogenized meat was hydrolyzed with 5 mL of 50 mM sulfuric acid at 80 °C for 90 min to release sialic acids from glycoproteins and glycolipids. After hydrolysis, the mixture was cooled on ice and centrifuged at 6000× *g* for 10 min at 4 °C. The resulting supernatant, which contained the released sialic acids, was collected and filtered through a regenerated cellulose syringe filter (0.45 µm pore size).

The derivatization step involved reacting the filtered hydrolysate with an equal volume of 1,2-diamino-4,5-dimethoxybenzene dihydrochloride (DMB) reagent. This mixture was incubated at 60 °C for 2.5 h to promote the formation of fluorescent DMB–sialic acid derivatives. The samples were analyzed using a Waters Alliance 2695 HPLC equipped with a fluorescence detector (Waters Corporation, Framingham, MA, USA). The separation of Neu5Ac and Neu5Gc was achieved using a C18 Synergi column (4 µm, 250 × 4.6 mm, Phenomenex Inc., Torrance, CA, USA). Fluorescence detection was performed using an excitation wavelength of 343 nm and an emission wavelength of 447 nm, allowing for the specific detection of DMB-derivatized Neu5Ac and Neu5Gc.

Neu5Ac and Neu5Gc peaks were identified by comparing their retention times to those of corresponding standard solutions. Data were referred as µg/g of meat.

### 3.5. Statistical Analysis

All data were analyzed using analysis of variance (ANOVA) with a one-way factorial model to assess differences between breeds. Meanwhile, a two-way factorial model, incorporating breeds and either times or muscles, was used to evaluate differences in maturation time or between the LTm and SMm of the HF and CN breeds. The general linear model (GLM) procedure in SAS (SAS Institute Inc., Cary, NC, USA) was applied for the ANOVA. Significant differences between groups were determined using Tukey’s test, with a significance threshold set at *p* < 0.05.

Correlation analysis was performed to evaluate the strength and direction of the relationship between sialic acids (Neu5Ac and Neu5Gc) and the main chemical characteristics of muscle, such as fat content, fatty acids, protein content, and amino acid profiles. The Pearson correlation coefficient (r) was used to test the correlations between the chemical constituents. A *p*-value < 0.05 was considered statistically significant.

The variabilities of Neu5Ac and Neu5Gc are reported in Supplementary Figure S1a,b.

## 4. Conclusions

This study highlights the wide variability in sialic acid content among different cattle breeds, particularly in Neu5Gc and Neu5Ac levels and their ratio, as well as differences between muscle types. The results demonstrate that Neu5Gc content varies significantly across breeds, with the highest levels observed in the Holstein Friesian and the lowest in the Piedmontese, followed by Limousin cattle. In general, specialized beef breeds exhibit lower Neu5Gc levels compared to dairy breeds. This extensive variability suggests that genetic background plays a crucial role in Neu5Gc accumulation, highlighting potential opportunities for genetic improvement in this trait. It is also important to acknowledge the favorable Neu5Ac content found in the meat of almost all the examined breeds, particularly in Piedmontese cattle. The dietary significance of Neu5Ac should not be overlooked, as it is an essential sialic acid that can only be obtained through the consumption of animal-derived products. The Neu5Ac/Neu5Gc ratio, a key factor in evaluating the nutritional quality of beef, also showed notable variability. Piedmontese cattle exhibited the highest ratio, surpassing the recommended dietary threshold of 5:1, suggesting that it may be a healthier dietary option within the beef sector. However, the Maremmana and Limousin breeds also demonstrated satisfactory Neu5Ac/Neu5Gc ratios, further emphasizing breed-specific differences in sialic acid composition. In addition to breed-related differences, this study also identified variability between muscles. While no statistically significant differences were observed, individual variation was evident, underscoring the complexity of factors influencing sialic acid composition in beef. Given the potential health implications associated with Neu5Gc intake, further research should explore strategies to reduce Neu5Gc content through selective breeding, dietary interventions, or processing techniques. Additionally, investigating the biochemical mechanisms underlying muscle-specific differences in sialic acid metabolism could provide further insights into optimizing meat quality for consumer health.

## Figures and Tables

**Table 1:** The percentage of fat, principal groups of fatty acids and protein, and principal classes of amino acids in the *Longissimus thoracis* of different beef breeds.

Breeds	N°	Fat%	SFA	MUFA	PUFA	Protein%	EAA%	BAA%	PAA%
HF	6	2.85 ^a^	46.51 ^a^	39.68	13.81 ^bc^	21.15 ^bc^	44.16	16.83 ^ab^	40.62 ^a^
RP	6	2.01 ^c^	45.77 ^ab^	40.97	13.26 ^c^	22.24 ^a^	42.58	16.31 ^b^	38.64 ^b^
MM	6	2.32 ^bc^	43.58 ^b^	39.67	16.74 ^b^	21.51 ^b^	44.36	18.21 ^a^	36.19 ^c^
CN	6	2.90 ^a^	44.16 ^b^	38.49	17.35 ^ab^	20.83 ^c^	44.10	18.06 ^a^	39.76 ^ab^
CH	6	2.44 ^ab^	45.72 ^ab^	40.94	13.31 ^c^	21.48 ^b^	43.09	15.49 ^b^	38.12 ^b^
LM	6	2.09 ^bc^	41.90 ^bc^	40.84	17.27 ^ab^	21.57 ^b^	42.99	15.61 ^b^	35.69 ^d^
PD	6	1.43 ^d^	40.22 ^c^	38.61	21.17 ^a^	22.44 ^a^	42.76	15.54 ^b^	40.62 ^a^
RMSE		0.52	2.30	2.93	3.89	0.49	2.02	1.33	1.63
*p* value		<0.001	0.002	0.010	0.003	<0.001	0.438	<0.001	<0.001

^a,b,c,d^ = different letters indicate significant differences as per *p* < 0.05; RMSE = root mean square error. Breeds: HF = Italian Holstein Friesian; RP = Italian Red Pied; MM = Maremmana; CN = Chianina; CH = Charolais; LM = Limousine; PD = Piemontese; N = animal number; SFA = saturated fatty acids; MUFA = monounsaturated fatty acids, PUFA= polyunsaturated fatty acids; EAA = essential amino acids; BAA = branch amino acids; PAA = polar amino acids.

**Table 2:** Content of N-acetylneuraminic acid (Neu5Ac) and N-glycolylneuraminic acid (Neu5Gc) and their ratios in the *Longissimus thoracis* of different beef breeds.

Breeds	N	Neu5Gc μg/g	Neu5Ac μg/g	Neu5Ac/Neu5Gc
HF	6	61.02 ^a^	110.34 ^a^	1.81 ^c^
RP	6	46.80 ^b^	90.45 ^bc^	1.93 ^c^
MM	6	31.09 ^c^	106.35 ^ab^	3.48 ^b^
CN	6	50.12 ^b^	103.82 ^ab^	2.07 ^c^
CH	6	47.40 ^b^	93.50 ^bc^	1.97 ^c^
LM	6	25.78 ^c^	81.25 ^c^	3.15 ^b^
PD	6	20.87 ^c^	112.99 ^a^	5.49 ^a^
RMSE		10.54	17.37	0.85
*p* value		<0.001	0.030	<0.001

^a,b,c^ = different letters indicate significant differences as per *p* < 0.05; RMSE = root mean square error. Breeds: HF = Italian Holstein Friesian; RP = Italian Red Pied; MM = Maremmana; CN = Chianina; CH = Charolais; LM = Limousine; PD = Piemontese; N = animal number.

**Table 3:** Correlation coefficients between sialic acids and chemical parameters.

	Fat%	SFA%	MUFA%	PUFA%	Protein%	EAA%	BAA%	PAA%
Neu5Gc	0.34 *	0.51 **	ns	−0.42 **	−0.37 *	ns	ns	ns
Neu5AC	ns	ns	ns	ns	ns	0.35 *	ns	0.44 **
Neu5Ac/Neu5GC	ns	−0.49 **	ns	0.46 **	ns	ns	ns	ns

Significant differences for each correlation coefficient “r”, with ns = not significant, * *p* < 0.05, ** *p* < 0.01, SFA = saturated fatty acids; MUFA = monounsaturated fatty acids; PUFA = polyunsaturated fatty acids; EAA = essential amino acids; BAA = branch amino acids; PAA = polar amino acids.

## Data Availability

Data presented in this article can be available at request.

## Supplementary Materials

The following supporting information can be downloaded at https://www.mdpi.com/article/10.3390/molecules30030710/s1: Table S1. Content of amino acids (expressed in % of total amino acids) in different beef breeds. Table S2. Content of N-acetylneuraminic acid (Neu5Ac) and N-glycolylneuraminic acid (Neu5Gc) in two different muscles and two different aging times of two breeds. Figure S1a,b. Distribution of Neu5Gc and Neu5Ac in different breeds: HF = Italian Holstein Friesian; RP = Italian Red Pied; CN = Chianina; MM = Maremmana; CH = Charolaise; LM = Limousine; PD = Piemontese.
