# Supplementary material for: Variability of Sialic Acids in Beef Breeds and Nutritional Implications in Red Meat
Source: Molecules. 2025 Feb 5;30(3):710. doi: 10.3390/molecules30030710 (PMC11821032; doi:10.3390/molecules30030710)
Supplement: Supplementary file 1 [file molecules-30-00710-s001.zip › molecules-3398688-supplementary.pdf]

Table S1. Contet of amino acids (expressed in % of total aminoacids) in different beef breads.

%	HF	RP	MM	CN	CH	LM	PD	P value	RMSE
**Asp**	9.33ab	9.65a	8.15b	9.29ab	8.39b	8.63b	9.81a	***	0.88
**Ser**	4.93a	4.61ab	3.58c	4.58ab	4.29b	4.64ab	4.44ab	***	0.55
**Glu**	13.59	12.81	12.25	14.25	13.87	12.19	14.77	ns	2.27
**Gly**	5.66ab	5.78a	5.82a	5.46ab	4.89b	5.54ab	5.40	**	0.84
**Ala**	5.27ab	6.30ab	5.38ab	5.13b	5.87ab	6.65a	5.66b	**	0.96
**Pro**	5.55b	6.37a	6.95a	5.56b	5.48b	7.10a	5.71b	***	0.89
**Cys**	1.63a	1.66a	0.75b	1.34ab	1.72a	1.75a	1.87a	***	0.59
**Tyr**	4.16ab	3.93b	4.52a	4.29a	4.60a	3.88b	3.83b	**	0.48
**Arg**	5.71	6.30	7.29	6.00	7.81	6.62	5.64	ns	2.03
**His**	3.91b	3.76b	4.72a	3.57b	3.74b	5.37a	3.98b	**	0.79
**Thr**	6.97a	5.97ab	6.94a	6.01ab	5.25b	4.59b	5.90ab	***	1.23
**Val**	4.11b	3.95b	5.55a	4.40ab	3.42b	3.27b	3.41b	**	1.21
**Met**	3.70	4.04	4.42	4.15	4.26	4.65	3.93	ns	0.97
**Lys**	8.44a	8.04a	5.97b	7.30a	8.72a	8.15a	8.53	**	2.24
**Ile**	4.27ab	4.05ab	3.92b	4.75a	3.88b	3.72b	4.02ab	*	0.83
**Leu**	8.44	8.31	8.75	8.91	8.19	8.63	8.11	ns	0.95
**Phe**	4.32b	4.46b	5.10ab	5.01ab	5.62a	4.59b	4.89ab	**	0.77

a,b,c= different letters indicate significant differences in the row per $p<0.05$; p value= *$p<0.05$, **$p<0.005$, ***$p<0.001$; RMSE= Root means square error. Breeds: HF= Italian Holstein Friesian, RP= Italian Red Pied, MM= Maremmana, CN= Chianina, CH= Charolais, LM= Limousine, PD= Piemontese; Asp= aspartic acid, Ser= serine, Glu= glutamic acid, Gly= glycine, Ala= alanine, Pro= proline, Cys= cystine, Tyr= tyrosine, Arg= arginine, His= histidine, Thr= threonine, Val= valine, Met= methionine, Lys= lysine, Ile= isoleucine, Leu= leucine, Phe= phenylalanine.

Table S2. Content in N-acetylneuraminic acid (Neu5Ac) and N-glycolylneuraminic acid (Neu5Gc) in two different muscles and two different aging times of two breeds.

	HF LTm	HL SMm		CN LTm	CN SMm		RMSE
**Neu5Gc mg/g**	61.02	54.38	ns	50.12	43.98	ns	15.72
**Neu5Ac mg/g**	110.34	120.38	ns	103.82	116.60	ns	20.14
	HF 1h	HF 10d		CN 1h	CN 10d		
**Neu5Gc mg/g**	53.11	61.02	ns	48.14	50.12	ns	11.02
**Neu5Ac mg/g**	102.85	110.34	ns	101.36	103.82	ns	18.41

HF= Italina Holstein Friesian, CN= Chianina; LTm= *Longissimus thoracis* muscle; SMm= *Semimembranosus* muscle; 1h= one hour after slaughter; 10d= ten days after slaughter.

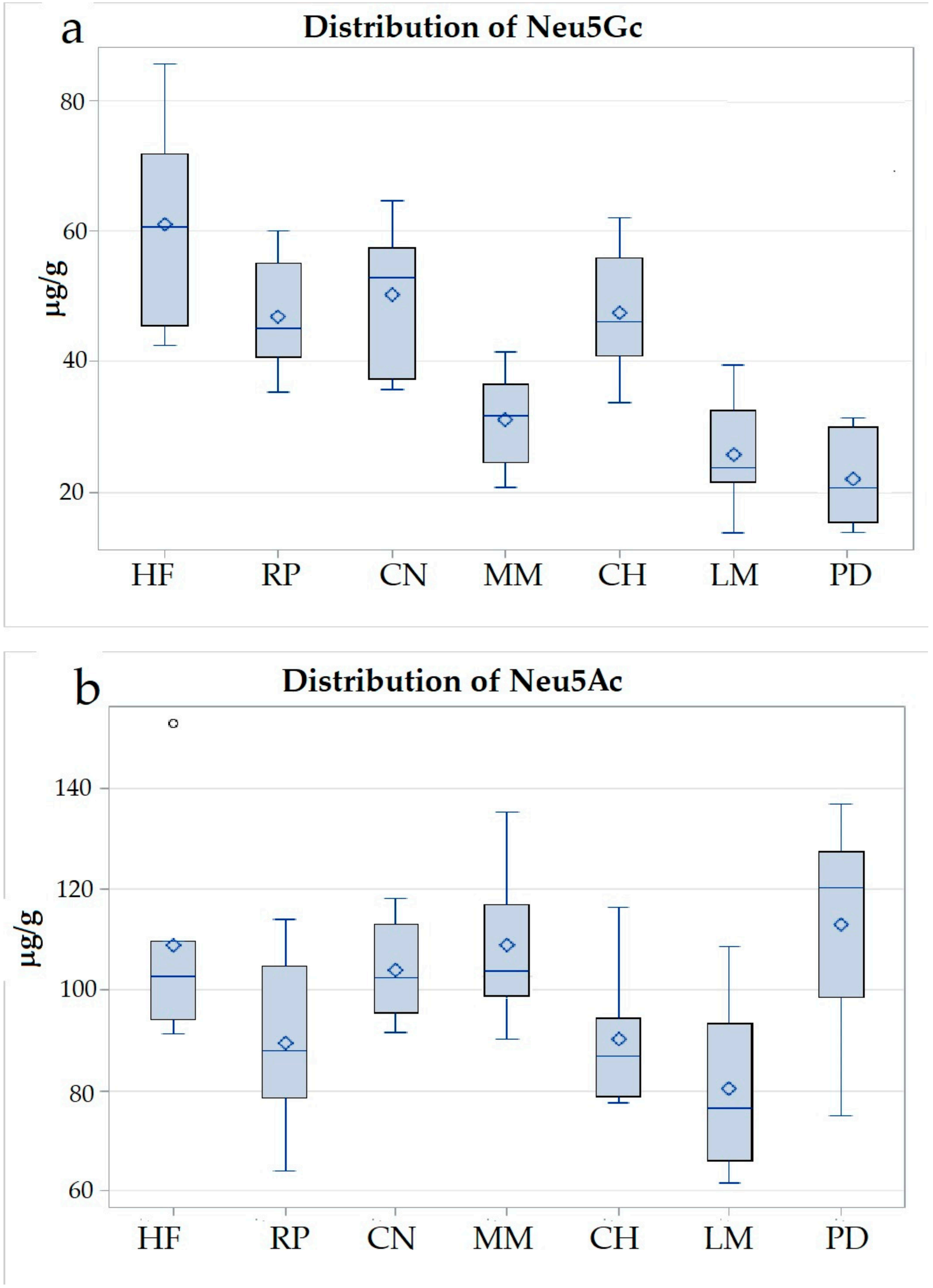

Figure S1 a and b: Distribution of Neu5Gc (a) and Neu5Ac (b) in different breeds. HF= Italian Holstein Friesian, RP= Italian Red Pied, CN= Chianina, MM= Maremmana, CH= Charolaise, LM= Limousine, PD= Piemontese.
